# Hemato-biochemical and hormonal profiles in post-partum water buffaloes (*Bubalus bubalis*)

**DOI:** 10.14202/vetworld.2015.512-517

**Published:** 2015-04-18

**Authors:** Sunil Kumar, A. K. Balhara, Rajesh Kumar, Naresh Kumar, Lukumoni Buragohain, Daoharu Baro, R. K. Sharma, S. K. Phulia, Inderjeet Singh

**Affiliations:** 1 Division of Animal Reproduction, Indian Veterinary Research Institute, Izatnagar, Uttar Pradesh, India; 2 Division of Animal Physiology and Reproduction, Central Institute for Research on Buffalo, Hisar, Haryana, India; 3 Department of Biochemistry, College of Basic Sciences and Humanities, CCS Haryana Agricultural University, Haryana, India; 4 Department of Animal Biotechnology, College of Veterinary and Animal Science, Lala Lajpat Rai University of Veterinary and Animal Sciences, Haryana, India

**Keywords:** biochemical, buffalo, follicular fluid, hematology, ovum pick up

## Abstract

**Aim:**

The objective of the present study was to compare serum as well as follicular fluid (FF) biochemical and hormonal profiles along with hematological parameters in postpartum estrus, anestrus, and cystic buffaloes.

**Materials and Methods:**

Postpartum buffaloes were selected in three different groups (within 40-60 days of parturition at estrus-Group-I, postpartum >90 days at anestrum-Group-II, and postpartum cystic buffaloes in Group III). The animals selected were examined for follicular wave dynamics by routine trans-rectal ultrasonography and FF was collected by transvaginal ultrasound-guided ovum pick up technique. All hematological and biochemical parameters were analyzed by automatic analyzers while hormonal profiles analyzed by commercially available ELISA kits.

**Results:**

In the present investigation, estrum and anestrum animal differ significantly in hemoglobin levels. Serum estradiol differs significantly in estrus and anestrus while no significant difference in progesterone concentration was noted among all three stages. The results of our study suggest that significant higher increase in total protein (TP), calcium and glucose values in estrum while urea, aspartate aminotransferase, alanine aminotransferase, lactate dehydrogenase significantly higher in anestrum animals.

**Conclusion:**

The conclusion of the present study is that TP and albumin, calcium, urea, glucose affects oocyte development and quality.

## Introduction

Among the livestock, Buffalo (*Bubalus bubalis*), the long-time ruminant animal contributing to the integrated farming systems, as a source of draft power, transportation, on-farm manure, meat, milk and livelihood of the farmers [1]. Buffaloes are important livestock resource for the local economy because of its high milk production and high demand around the world. Interest in buffalo breeding has tremendously increased worldwide due to the fundamental role played by the species in many climatically disadvantaged agricultural systems [2]. Such adverse conditions may affect the health and reproductive efficiency of animals. Animal health can be defined as the absence of disease determined by clinical examinations combined with various diagnostic tests [3]. Hematological and serum biochemical reference values are used to diagnose systemic diseases. To investigate reproductive performance, follicular fluid (FF) serves a good sample particularly in reference to reproductive endocrinology [4]. FF, in part, is a transudate of serum, as surrounding cell layers permit the free diffusion of proteins of up to 500 kDa [5]. It is a complex of restricted components of serum and follicular synthesized secretions [6]. The permeability of the follicular wall to water and biochemical components originating from blood may markedly alter the composition of FF. Most of literature in buffalo FF analysis is based on ovarian FF collected from morbid genitalia [7].

In the present study, FF was aspirated by *ex vivo* puncture of ovarian follicles. So the present study was planned with the objective to compare, both in serum as well as FF, biochemical and hormonal profiles along with hematological parameters in postpartum estrum, anestrus, and cystic buffaloes.

## Materials and Methods

### Ethical approval

The present investigation has been conducted with approval of Institutional Animal Ethics Committee, CIRB, Hisar.

### Experimental animals

**Figure-1:**
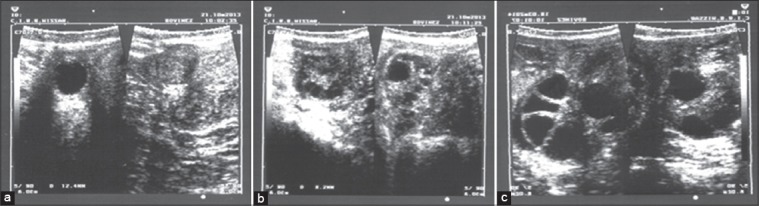
Ultrasonographic images of ovarian follicles in estrus (a), anestrum (b), cystic (c) animals

The present study was carried out at the Animal Farm of the Central Institute for Research on Buffaloes, Hisar, India. Postpartum buffaloes were selected in three different groups (within 40-60 days of parturition at estrus-Group-I, postpartum >90 days at anestrum-Group-II, and postpartum cystic buffaloes in Group III). Blood was collected from 10 animals in each group while FF was collected from 6 animals in Group-I and III but from 9 animals in Group-II. The animals selected were examined for follicular wave dynamics by routine trans-rectal ultrasonography (USG) with ‘B mode’ ultrasound scanner (Just Vision 200, Model SSA 320A, Toshiba, Japan) equipped with an intra-operative 7.0 MHz micro-convex multi-frequency transducer. Mean (mm) diameters of the largest follicle (Figure-1) at the time of FF Collection in Group-I (14.05±0.64), Group-II (12.42±0.73) and Group-III (23.65±2.25) was observed. FF collected from morphologically dominant follicle by transvaginal ultrasound-guided ovum pick up technique. Approximately 6 ml of venous blood sample was collected in Vacutainer^®^ serum-separator tube and 6 ml in K2 ethylenediaminetetraacetic acid tube (BD Biosciences, USA) by Jugular venipuncture from experimental animals simultaneously just before the time of FF collection. Blood was immediately mixed with the gel coating inside the tubes by inverting at least 5 times and allowed to stand at room temperature for 1 h. Serum was separated by centrifugation at 1500 rpm for 20 min and transferred in clean pre-sterilized cryovials and stored at −20 until analysis.

### Hematological analysis

Hematology was carried using automatic veterinary scan hematological analyzer directly after the samples were received by the research laboratory and within 30 min after samples were collected. Hematological variables measured were red blood cell count, hemoglobin concentration (Hb), plateletcrit, total leucocyte count (TLC), lymphocytes (%), monocytes (%), neutrophils (%), eosinophils (%), basophils (%) and platelets.

### Biochemical analysis

Biochemical analysis was carried out using automatic Biochemical analyzer (tulip biochemical analyzer, JAPAN). The serum and FF biochemical parameters were measured as: Aspartate aminotransferase (AST) (U/L), lactate dehydrogenase (LDH) (U/L), calcium (mg/dl), alanine aminotransferase (ALT) (U/L), creatinine (mg/dl), cholesterol (mg/dl), triglycerides (TG) (mg/dl), total protein (TP) (g/dl), phosphorus (mg/dl), total bilirubin (mg/dl), Glu (mg/dl), albumin (g/dl), chloride (mmol/l) and alkaline phosphatase (ALP) (U/L). ALP was carried only in FF.

### Hormonal analysis

Estradiol and progesterone were measured both in serum as well as FF using commercially available ELISA kits. Protocol for assay was as per manufacturer’s instructions.

### Statistical analysis

Data from the present study were compared with established reference values for serum biochemical [8] and hematological [9] constituents of cows. The FF values of the present investigation have been compared with published values in literature. Mean values and standard error has been derived using one-way ANOVA with Duncan test.

## Results

In hematological analysis, estrum and anestrum animals differ significantly in hemoglobin levels (p<0.05) among all parameters. No significant difference was observed in cystic animals. FF biochemical parameters i.e. aspartate aminotransferase (p<0.05), ALT (p<0.05), calcium (p<0.01), TP (p<0.05), albumin (p<0.05), glucose (p<0.05) while serum biochemical parameters i.e. LDH (p<0.05), Urea (p<0.05) and TP (p<0.05) has been found to vary significantly among different groups. Serum estradiol differs (p<0.05) significantly in estrum and anestrum animals while no significant difference in progesterone concentration in both serum as well as was not noted among all three groups. Wide variations have been found in serum as well as FF estradiol values in anestrus animals indicating continuous growth, atresia and regression of ovarian follicles. The results of the hematological, biochemical and hormonal analysis are summarized in Tables-1-4.

**Table-1:** Hematological parameters.

Group	I	II	III	Feldman, Zinkl and Jain (2000)
Hb (g%)	13.34±0.43^b^	11.38±0.56^a^	12.7311±0.87^a^	8-15
PCT (%)	0.31±0.02882	0.33±0.02	0.312±0.03	0.018-0.21
TEC (×10^12^/L)	7.95±0.40	6.85±0.37	7.97±0.43	5.00-10.00
TLC (×10^9^/L)	14.74±1.65	11.60±1.01	17.06±3.36	4.00-12.00
Ly (%)	48.49±4.34	41.81±2.74	49.27±4.06	45.00-75.00
Mo (%)	5.5±1.09	4.76±0.72	4.31±0.95	2.00-7.00
Ne (%)	45.4±4.03	52.69±2.5	44.53±3.27	15.00-45.00
Eo (%)	0.49±0.14	0.70±0.19	1.7666±1.03	0.00-20.00
Ba (%)	0.07±0.027	0.016±0.01	0.1±0.05	0.00-2.00
Platlets	323.6±27.33	343.25±25.95	320.44±26.43	295.61-328.08

Hb=Hemoglobin concentration, PCT=Plateletcrit, TEC=Total erythrocyte count, TLC=Total leucocyte count

**Table-2:** FF biochemical parameters.

Group	I	II	III	Reference^x^
Chloride (mmol/l)	111.91±10.82	101.81±5.29	114.06±2.97	90.74±6.86
Calcium (mg/dl)	5.37±0.58^b^	2.33±0.58^a^	4.67±0.7^b^	3.76-14.77
LDH (U/L)	1781.6±90.19	2061.8±180.8	1711.9±180.69	-
AST (U/L)	77.35±13.35^a^	127.09±6.72^b^	52.28±6.05^a^	-
ALT (U/L)	75.78±8.5^b^	89.43±7.71^b^	25.65±1.86^a^	-
Creatinine (mg/dl)	1.55±0.25	2.0±0.18	1.81±0.20	0.74±0.32
Urea (mg/dl)	48.75±4.25	58.5±6.75	50.7±5.018	36±2.4
Cholesterol (mg/dl)	101.68±7.8	123.4±13.37	128.02±14.16	108.19±5.25
TG (mmol/l)	46.35±2.0	45.02±1.73	44.06±1.39	41.55±10.93
TP (g/dl)	7.9±0.4^b^	6.94±0.19^a^	6.3±0.47^a^	4.34-14.66
Phosphorus (mg/dl)	3.65±0.21	3.36±0.29	3.2±0.26	1.81-7.85
Total bilirubin (mg/dl)	0.15±0.08	0.08±0.01	0.093±0.018	1.39-1.67
Albumin (g/dl)	3.92±0.3^b^	2.99±0.33^a^	2.7±0.10^a^	4.10±0.14
Glucose (mg/dl	72.25±4.11^b^	68.4±5.03^a^	88.62±5.6^a^	42.95±4.54
ALP (U/L)	254.68±62.74	201.6±23.76	308.65±30.85	217.7±17.8^x^

X=Based on published values.

a,b differ significantly with p<0.05, LDH=Lactate dehydrogenase, AST=Aspartate aminotransferase, ALT=Alanine aminotransferase, TP=Total protein, TG=Triglycerides

**Table-3:** Serum biochemical parameters.

Group	I	II	III	Kaneko, Harvey & Brass (1997)
Chloride (mmol/l)	149.9±4.34	158.88±3.091	55.29±7.19	97-111
Calcium (mg/dl)	12.9±0.36	12.45±0.63	13.22±0.46	9.7-12.4
LDH (U/L)	1561.55±95.78^a^	1964.51±103.28^b^	1814.22±101.80^a^	692-1445
AST (U/L)	149.95±13.29	174.21±10.451	58.07±9.84	78.0-132.0
ALT (U/L)	65.62±5.25	76.58±6.61	59.6±8.79	11.0-40.0
Creatinine (mg/dl)	1.87±0.11	1.819±0.18	1.63±0.22	1.0-2.0
Urea (mg/dl)	39.30±4.2^a^	47.05±4.8186^b^	29.93±5.5^a^	20-45^x^
Cholesterol (mg/dl)	108.16±7.43	138.51±13.85	105.84±13.05	80-120
TG (mmol/l)	44.30±1.11	42.4±1.54	41.72±1.26	46.49-52.32
TP (g/dl)	8.21±0.37^b^	6.96±0.22^a^	7.17±0.23^a^	6.0-14.2^x^
Phosphorus (mg/dl)	4±0.20	4.12±0.36	3.61±0.19	5.60-6.50
Total bilirubin (mg/dl)	0.16±0.03	0.158±0.037	0.16±0.03	1.0-2.0
Albumin (g/dl)	3.20±0.06	3.48±0.15	3.19±0.09	3.0-5.1^x^
Glucose (mg/dl)	65.27±5.07	65.7±5.36	71.2±5.05	45-75

X=Based on published values a and b differ significantly, LDH=Lactate dehydrogenase, AST=Aspartate aminotransferase, ALT=Alanine aminotransferase, TP=Total protein, TG=Triglycerides

**Table-4:** Mean concentration of P4 and E2 in serum and FF.

Group	Progesterone (ng/ml)		Estradiol (pg/ml)	
	
	FF	Serum	FF	Serum
I	16.28±4.08	0.31±0.04	359.3±20.19	105.84±34.12^a^
II	18.32±2.24	0.49±0.06	295.14±36.35	45.70±5.45^b^
III	16.55±3.09	0.38±0.04	258.97±64.42	73±12.9
Reference^x^	0.02-27.46	0.1-6	224.33±42.12	20-251

X=based on published values a and b differ significantly, FF=Follicular fluid

## Discussion

Most of previous reports on biochemical and hormonal estimation in FF are from slaughtered animal’s ovaries. To our best knowledge, this is the first report on buffalo’s FF biochemical and steroidal hormonal millue. Most of the hematological and biochemical parameters have been found within normal ranges as per earlier reports. It showed the normal physiological body conditions of selected animals under the study. In hematological analysis, only a significantly (p<0.05) higher hemoglobin has been found. Because hemoglobin is a conjugated transport protein, higher values in estrus animals may be due to increased protein synthesis which in the present investigation has been found significantly higher in estrus animals. Considering ovulation as an inflammatory event, consequently it should alter TLC at estrus but in the present study no significant difference was found. It may be due to the fact that ovulatory inflammation is a much localized inflammation that is why not altering TLC systemically.

Almost all biochemical parameters resulted higher values as compared to FF values. It indicates that FF is results from ultra-filtration of serum through follicular basement membrane. But estradiol, progesterone, glucose and urea are measured in higher concentration as compared to serum indicating that they are produced by the follicle. Thus, FF can’t be solely regarded as a transudate. From the present study, this inference can be drawn that FF is a combination of serum transudate and secretions of follicular cells.

In the present study, it was also observed that the TP and albumin concentrations of serum as well as FF were significantly higher in estrus which are in agreement with those found in buffaloes [10]. The increased amount of protein in FF at estrus may be attributed due to increase in permeability of blood vessels across the basement membrane. The high correlation between TP and albumin content in FF and serum suggest that a substantial part of protein contents in FF originate from the serum. At estrus, during ovulation, some FF containing albumin is picked by fimbria which play a role in sperm capacitation by inducing cholesterol efflux. This may be one explanation for an increase in albumin at estrus. The present findings demonstrate that FF concentrations of glucose were significantly higher (p<0.05) in estrum animals than anestrus and cystic animals. Glucose serves as one of the main biochemical metabolites affecting oocytes growth and development, varied significantly during follicle development [11]. In line with findings of the present research, Leroy *et al*. [12], Tabatabaei *et al*. [13] showed that as follicle size got enlarged at estrus, glucose concentration was increased simultaneous with increase in follicle size and follicles size might be due to increased FF secretion within dominant follicles [14]. It is likely that larger follicles in estrus have less glucose metabolism (per FF volume) than small ones in anestrus, in turn results in less uptake of FF glucose by granulosa cells [15]. This implies that the principal source of FF glucose is blood, and very little glucose is synthesized locally by the granulosa cells of follicles. The hypoglycemic state in buffaloes reduced the hypothalamic-hypophyseal-ovarian axis signal transmission leading to anestrus condition [16]. But, in the present study, it was observed that the glucose concentrations of serum were significantly lesser (p<0.05) than FF. These results are not in agreement to those reported by [11] in dairy cows, [17] in buffaloes, who found that the glucose concentrations in blood plasma were significantly higher (p<0.05) than FF. This is in contradiction to statement that the principal source of FF glucose is blood, and very little glucose is synthesized locally by the granulosa cells of follicles. The plasma triglyceride levels (mg/dl) between anestrus and regular cyclic buffaloes reported no significant variation and had been reported that serum TG concentration is not related to resumption of postpartum ovarian cyclicity in cattle [18]. Cholesterol is the precursor for steroid synthesis and the FF contained only high-density lipoprotein, therefore, the vascular granulose cells of the follicles totally depended on the cholesterol from high-density lipoprotein, which was derives from the blood plasma by crossing the basement membrane of granulose cells [19].

The result of the present study also indicated that FF AST, ALT and serum LDH concentrations in anestrus were significantly higher (p<0.05) than estrus, while the concentrations of calcium in estrus were significantly higher (p<0.01) than anestrus and cystic animals. Higher LDH, AST and ALT concentrations indicate greater body metabolic load in anestrus than in estrus animals while lower calcium values in anestus indicate their requirement in metabolic pathways involved in ovulation in association with protein kinases.

The increased concentration of Ca^2+^ may be related to steroidogenic capability of the estrual follicle, in that Ca^2+^ plays an important role in the gonadotropic regulation of ovarian steroidogenesis. Lower levels of calcium may be attributed to consequences to absence of luteinizing hormone (LH) surge in anestrus because LH surge causes adenylate cyclise mediated calcium ion influx [20]. This may be one of the reasons for atresia, regression or anovulation of the dominant follicle in anestrus animals because the calcium ions affect oocyte development competence which is essential for meiotic resumption and subsequently ovulation. LDH, AST, and ALT are liver-specific enzymes, so their correlation with leptin in relation to postpartum anestrus provides a scope of further study. Inter- relationships of increased albumin and TP concentrations in FF with increased LDH and alkaline phosphatase activity seem to indicate that a number of biochemical changes may relate to the atresia process in anovulatory follicles and provide useful tools for understanding follicular regression in anestrus animals.

Serum urea has been found significantly (p<0.05) higher in anestrus animals than in estrus and cystic animals indicating metabolic stress in affected animals. Higher urea concentration affects developing oocytes quality so that too urea levels in FF interrupts blastocyst formation, lowers pH in reproduction system tissues and uterus unsuitable for fetus [21]. Low urea concentration leads to desirable oocytes development as follicles diameter enlarges [22]. Results of the present study were confirmed by findings of Leroy *et al*. [12], they measured various urea concentrations in different sized follicles and concluded that as follicles enlarged (i.e. estrus), urea concentration was decreased accordingly [21,22]. In hormonal profiles, a wide variation has been observed in serum as well as FF estradiol concentration indicating continuous follicular growth and atresia in anestrus animals [23]. Lower levels of estradiol may be due lack of LH support or vice-versa in cystic animals [24]. Progesterone levels are in accordance with published values in literature indicating lower concentration at the time of estrus [25]. It is thought that the turnover of ovarian steroids is rapid and that the ovary does not have large stores of them; thus FF concentrations of steroidal hormones may be a true index of follicular endocrine well-being. Indications are that the steroid content of FF varies according to the size, stage of development and stage of the estrous cycle.

## Conclusion

The present study was conducted to correlate the roles of hematological, biochemical and hormonal parameters in relation to estrus, anestrus and cystic reproductive stages in postpartum buffaloes. The conclusion of the present study is that calcium, glucose, and urea might have played an important role in follicular dynamics in estrus and anestrus animals by affecting the ovulatory mechanisms and oocyte quality. Increased TP and albumin in estrus might be due to increased physiological needs and requirement for capacitation. Progesterone remains more or less within the same range in estrus, anestrus animals while higher values of progesterone in the FF as compared to estradiol indicate that cystic follicles are estrogenically inactive. The significant positive correlation between follicular estrogen and progesterone found in the present work is consistent with possibility that follicular progesterone serves as a precursor to androgen and subsequently estrogen production by follicles of buffaloes. Increased knowledge of biochemical changes in relation to endocrine and structural alterations in growing/estrual and atretic follicles will lead to an understanding of the complexity of events surrounding the development of the follicle.

## Authors’ Contributions

IJS and AKB designed the work, SK conducted and analyzed the study, RKS and SKP helped in Ultrasonography and Ovum Pick Up. RK, LB, DB and NK helped in sampling and processing. SK and AKB drafted and revised the manuscript. All authors read and finalized the manuscript.
